# Enhanced Electrical Properties and Impact Strength of Phenolic Formaldehyde Resin Using Silanized Graphene and Ionic Liquid

**DOI:** 10.1021/acsomega.3c05198

**Published:** 2023-12-19

**Authors:** Yan-Chun Li, Seul-Yi Lee, Hong Wang, Fan-Long Jin, Soo-Jin Park

**Affiliations:** † Department of Chemistry and Pharmaceutical Engineering, Jilin Institute of Chemical Technology, Jilin City 132022, People’s Republic of China; ‡ Department of Chemistry, Inha University, Inharo 100, Incheon 22212, South Korea; § Institute of Petrochemical Technology, Jilin Institute of Chemical Technology, Jilin City 132022, People’s Republic of China; ∥ Department of Polymer Materials, Jilin Institute of Chemical Technology, Jilin City 132022, People’s Republic of China

## Abstract

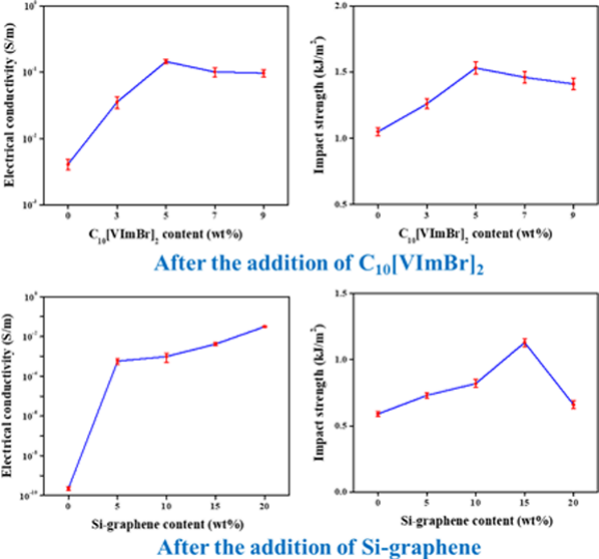

In this study, to improve the electrical properties and impact strength of phenolic formaldehyde (PF) resin, PF-based composites were prepared by mixing graphene and the ionic liquid 3-decyl-bis(1-vinyl-1*H*-imidazole-3-ium-bromide) (C_10_[VImBr]_2_) via hot blending and compression–curing processes. The graphene surface was modified using a silane coupling agent. The synergistic effect of graphene and C_10_[VImBr]_2_ on the electrical properties, electromagnetic shielding efficiency, thermal stability, impact strength, and morphology of PF/graphene and PF/graphene/C_10_[VImBr]_2_ composites was then investigated. It was found that the electrical conductivity of the composites significantly increased from 2.3 × 10^–10^ to 4.14 × 10^–3^ S/m with an increase in the graphene content from 0 to 15 wt %, increasing further to 0.145 S/m with the addition of 5 wt % C_10_[VImBr]_2_. The electromagnetic shielding efficiency of the composite increased from 4.70 to 28.64 dB with the addition of 15 wt % graphene, while the impact strength of the composites rose significantly from 0.59 to 1.13 kJ/m^2^ with an increase in the graphene content from 0 to 15 wt %, reaching 1.53 kJ/m^2^ with the addition of 5 wt % C_10_[VImBr]_2_. Scanning electron microscopy images of the PF/GNP/C_10_[VImBr]_2_ composites revealed a rough morphology with numerous microcracks.

## 1 Introduction

Because it is an outstanding flame retardant with good adhesion, high heat resistance, excellent mechanical properties, and straightforward processability, phenolic formaldehyde (PF) resin has been extensively used in a number of applications, including coatings, construction, transportation, electronics, and the automotive and aerospace industries.^1−5^ However, its intrinsically low electrical conductivity and high brittleness have limited its widespread use in other fields.^6−8^ Thus, in recent years, electrically conductive polymer composites have attracted considerable attention due to their lightweight, corrosion resistance, and easy processing.

Generally, electrically conductive polymer composites are prepared by dispersing an electrically conductive filler, such as a metal or a carbon nanomaterial, within a polymer matrix.^9−11^ As conductive fillers, metals such as silver, copper, and aluminum have several disadvantages, including high density and cost. In contrast, carbon nanomaterials, such as graphene and carbon nanotubes (CNTs), exhibit many advantages. Graphene has a two-dimensional planar sheet structure composed of sp^2^ hybridized carbon atoms with each carbon atom, possessing a free electron in the π orbital that can move freely within the lattice. The π-orbital contributes to the formation of a delocalized electron network, meaning that graphene has an extremely high electron mobility of up to 250,000 cm^2^/v·s at room temperature and a low resistivity of 10^–6^ Ω·cm.^12−15^ Graphene also has an ultrahigh specific surface area, remarkable mechanical properties, low density, and excellent chemical stability. These unique properties make graphene an ideal electrically conductive filler when seeking to improve the electrical conductivity of polymer composites.^16^ CNTs have a hollow, seamless tube-like structure consisting of cylindrical curled graphene sheets with a high aspect ratio. This unique structure leads to an intrinsic mobility that is as high as 100,000 cm^2^/V·s at room temperature, with a current carrying capacity as high as 10^9^ A/cm^2^.^17,18^ CNTs also have high thermal and electrical conductivity, excellent strength and modulus, good chemical stability, a large specific surface area, and a low density, making them particularly suitable as an electrically conductive filler for polymer composites.^19−21^

The development of functional composites based on carbon nanomaterials has attracted significant recent attention due to their functions and practicality. These composites can be used as active media for functional devices in high-frequency applications, such as electromagnetic shielding, antenna technology, and electromagnetic compatibility.^22−25^ However, previous studies have proven that graphene in a polymer matrix is not evenly dispersed and aggregates at relatively low volume fractions because of its large surface area and strong van der Waals forces.^26−28^ This has restricted the use of graphene in polymer composites. Therefore, the graphene surface is generally modified or functionalized using organic molecules to improve the compatibility between graphene and the polymer matrix.^29−31^

Ionic liquids (ILs), which are molten salts consisting of bulk organic cations and organic or inorganic anions, exhibit a number of attractive physical and chemical properties, such as low volatility, good compatibility, nonflammability, high thermal and chemical stability, and good electrical conductivity.^32,33^ ILs also have low toxicity, are recyclable, and are functionalizable, which is why they have been widely employed in electrochemistry, organic synthesis, chemical separation, and material preparation.^34,35^

To date, a variety of ILs have been synthesized and used to improve the electrical properties of polymer materials. For example, Ogoshi et al. prepared transparent ion-conductive IL–phenol resin hybrids via the in situ polymerization of phenol monomers in the presence of an IL.^36^ They reported that a transparent hybrid containing 20 wt % phenol resin had a high thermal stability and an ionic conductivity of 1.0 × 10^3^ S/m at 30 °C. Guo et al. synthesized three ILs and used them as solvents for corn stalks during phenolic resin modification.^37^ The tensile strength and impact strength of the phenolic resin modified with the ILs improved from 3.28 MPa and 0.93 kJ/m^2^ to 9.36 MPa and 5.74 kJ/m^2^, respectively. Younesi-Kordkheili studied the properties of particleboard panels bonded with IL-treated lignin–phenol–glyoxal resin.^38^ The use of the IL-modified lignin not only led to a more rapid gelation time but also increased the viscosity, density, and solid content of the resulting resin, thus reducing the temperature required for curing. They subsequently investigated the physical and mechanical properties of plywood panels bonded with IL-modified lignin–phenol–formaldehyde resin,^39^ finding that the mechanical properties of the panels were significantly enhanced with an increase in the IL-modified lignin content from 0 to 20 wt %.

Li et al. synthesized animidazolium IL-modified phenolic resin (ILPR) that more effectively extracted the benzoylurea plant hormones thidiazuron and forchlorfenuron than unmodified phenolic resin due to the presence of imidazolium in the IL.^40^ Wang et al. also synthesized the IL 3-decyl-bis(1-vinyl-1*H*-imidazole-3-ium-bromide) (C_10_[VImBr]_2_) and used it to improve the electrical conductivity of PF/graphene composites.^41^ The electrical conductivity of the composites increased from 5.6 × 10^–3^ to 9.2 × 10^–2^ S/m when the IL content increased from 0 to 5 wt %. Yao et al. synthesized the IL 1,2-dimethyl-3-butylimidazole bromide salt and employed it in PF-based conductive materials.^42^ They reported that the thermal stability and impact strength of the PF/IL system increased with the addition of the IL, while its volume resistance significantly decreased from 1.02 × 10^9^ to 1.64 × 10^7^ Ω when the IL content rose from 0 to 1 wt %.

In the present study, PF-based composites with improved electrical properties and impact strength were prepared by combining graphene and the IL C_10_[VImBr]_2_ by using hot blending and compression–curing processes. The graphene surface was modified using a silane coupling agent. The synergistic effect of graphene and C_10_[VImBr]_2_ on the electrical properties, electromagnetic shielding efficiency, thermal stability, impact strength, and morphology of PF/graphene and PF/graphene/C_10_[VImBr]_2_ composites was also investigated.

## 2 Experimental Section

### 2.1 Materials

**Figure 1:**
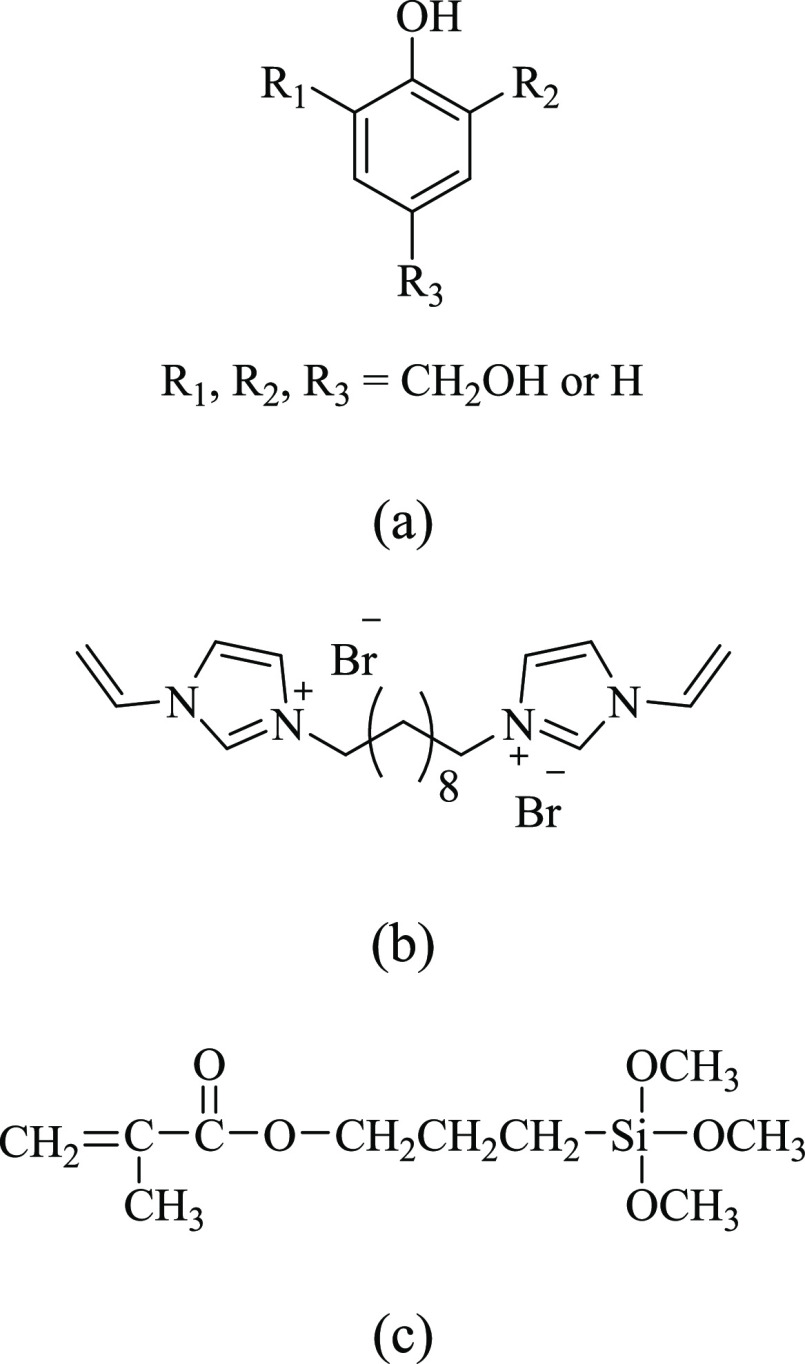
Chemical structures of (a) PF, (b) C_10_[VImBr]_2_, and (c) KH-570.

PF with a dynamic viscosity of 11,000 mPa-s was synthesized for use in the present study.^42^ Graphene was obtained from Yantai Sinagraphene Co., Ltd. (Yantai, Shandong, China), with a carbon content of 96 wt % and an electrical conductivity of 50,000 S/m. C_10_[VImBr]_2_ was synthesized following a route described in a previous report.^41^ The silane coupling agent γ-methacryloxypropyltrimethoxysilane (KH-570) was obtained from Sahn Chemical Co., Ltd. (Shanghai, China). Anhydrous ethanol was purchased from Tianjin Yongda Chemical Reagent Co., Ltd. (Tianjin, China). The chemical structures of PF, C_10_[VImBr]_2_, and KH-570 are presented in Figure 1.

### 2.2 Surface Modification of Graphene

To improve the dispersion of graphene within the PF matrix, the surface of graphene was modified by using the silane coupling agent. Graphene (25 g) was dispersed in anhydrous ethanol (400 mL) and then ultrasonically treated for 2 h to obtain a well-dispersed graphene solution. KH-570 (14 g) and water (16 mL) were then added to the graphene solution, and the mixture was heated to 55 °C and left to react for 1.5 h. After the reaction, the solution was vacuum-filtered, washed with deionized water to a neutral pH, and dried at 60 °C for 3 h in a vacuum oven to obtain the surface-modified graphene (hereafter, Si-graphene).

### 2.3 Preparation of the PF/Si-Graphene Composites

The PF/Si-graphene composites were prepared by using hot blending and compression–curing processes. The Si-graphene content in the PF/Si-graphene composite varied between 0 and 20 wt %. In the typical process, selected amounts of PF and Si-graphene were stirred at 60 °C for 30 min and then mixed at 25 °C for 30 min using a spin stirrer. The mixture was then heated to 145 °C in a vacuum oven and injected into a mold that had previously been sprayed with a mold-release agent. The mixture was then compression-cured at 145 °C under a pressure of 10 MPa for 30 min.

### 2.4 Preparation of the PF/Si-Graphene/C_10_[VImBr]_2_ Composites

The PF/Si-graphene/C_10_[VImBr]_2_ composites were synthesized using the same method used for the PF/Si-graphene composites. The Si-graphene content in the composites was 15 wt %, and the C_10_[VImBr]_2_ content varied between 0 and 9 wt %. In the typical process, selected amounts of PF, Si-graphene, and C_10_[VImBr]_2_ were stirred at 60 °C for 30 min and then mixed at 25 °C for 30 min using a spin stirrer. The mixture was then heated to 145 °C in a vacuum oven and injected into a mold, which had previously been sprayed with a mold-release agent. The mixture was then compression-cured at 145 °C under a pressure of 10 MPa for 30 min.

### 2.5 Characterization and Measurements

The pristine graphene, Si-graphene, and PF/Si-graphene composites and PF/Si-graphene/C_10_[VImBr]_2_ composites were characterized using a variety of analytical techniques. The functional groups present in the pristine graphene and Si-graphene were characterized using a Nicolet 6700 Fourier transform infrared (FTIR) spectrometer (Thermo Fisher Scientific), while the surface properties of pristine graphene and Si-graphene were evaluated using X-ray photoelectron spectroscopy (XPS; Thermo ESCALAB 250) with a monochromatic Al K_α_ source and a passing energy of 20 eV. The surface morphology of pristine graphene and Si-graphene was investigated via scanning electron microscopy (SEM; Zeiss, Gemini 500). Energy-dispersive X-ray spectroscopy (EDX) and SEM were conducted to verify the presence of Si on the graphene surface.

The electrical conductivities of the PF/Si-graphene and PF/Si-graphene/C_10_[VImBr]_2_ composites were measured at room temperature using a DC resistance tester (ZC-90) following the GB/T 24525-2009 standard. The size of the samples was 5 × 20 × 30 mm^3^. The electrical conductivity (σ) was calculated using eq 1
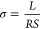
1where *L* and *S* are the thickness and cross-sectional area of the sample, respectively, and *R* is its measured resistivity. The overall electrical conductivity was determined by averaging the five experimental values.

The electromagnetic shielding efficiency of the composites was measured using a Vector network analyzer (E5071C) in the X-band frequency range of 2–18 GHz at room temperature following the GB/T 32596-2016 standard. The electromagnetic shielding efficiency (SE_T_) was calculated using eq 2
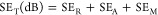
2where SE_R_, SE_A_, and SE_M_ are the reflection, absorption, and multiple internal reflection shielding efficiency, respectively. The electromagnetic shielding efficiency was determined by averaging three experimental values.

The thermal stability of the composites was investigated via thermogravimetric analysis (TGA; TA Instruments, Q50) at a temperature range of 30–800 °C and a scanning rate of 10 °C/min under a nitrogen atmosphere. In addition, the impact strength of the composites was measured by using an Izod impact tester (TP04G-AS1) in accordance with the GB/T 1843–2008 standard. The size of the samples for this test was 4 × 10 × 50 mm^3^. The impact strength was determined by averaging five experimental values. Finally, the morphology of pristine PF and the composites after the impact strength tests was examined using SEM (Zeiss, Gemini 500).

## 3 Results and Discussion

### 3.1 Characterization of Si-graphene

**Figure 2:**
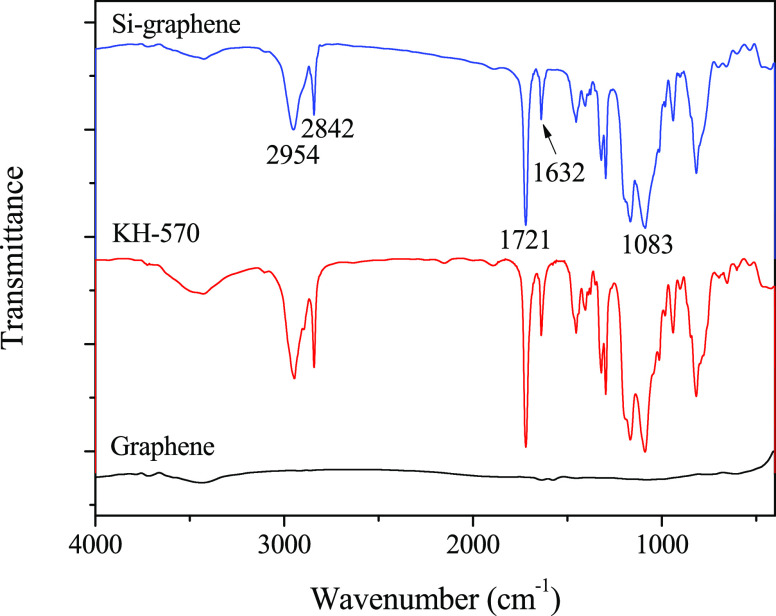
FTIR spectra for graphene, KH-570, and Si-graphene.

The surface of the graphene was modified using KH-570 as a silane coupling agent, and the structure of Si-graphene was then characterized. The changes in the functional groups before and after the surface modification of graphene were characterized using FTIR. Figure 2 presents the FTIR spectra for pristine graphene, KH-570, and Si-graphene. After surface modification, two characteristic absorption peaks appeared at 2914 and 2853 cm^–1^, which were assigned to C–H antisymmetric and symmetric stretching vibrations, respectively. Three characteristic absorption peaks also appeared at 1733, 1632, and 1083 cm^–1^, which were attributed to C=O telescopic vibrations, C=C stretching vibrations, and C–O–Si bonds, respectively. These results could be attributed to the introduction of organic functional groups, such as methylene, methyl, C=O, and C–O on the graphene surface due to the surface modification.^43^

**Figure 3:**
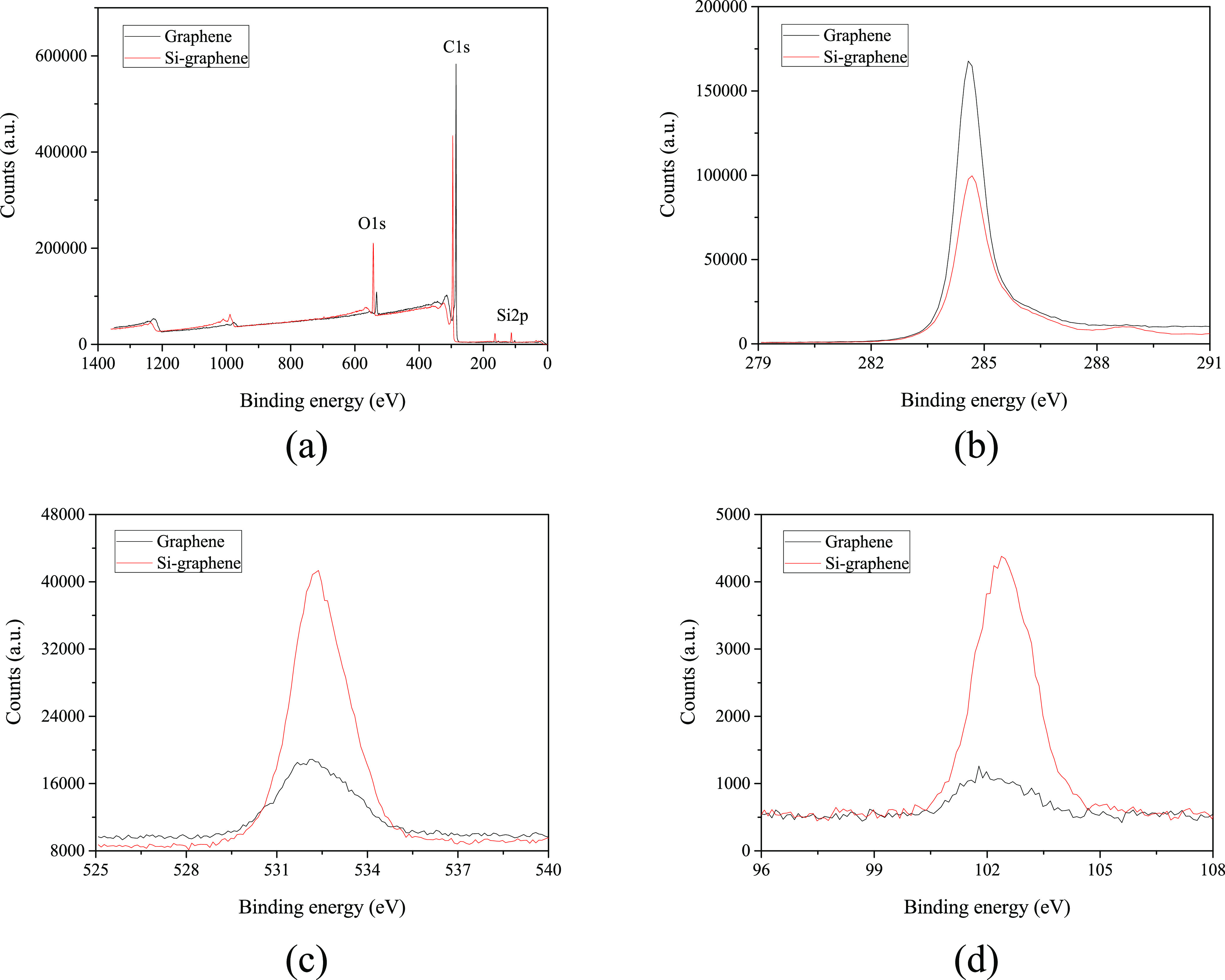
High-resolution XPS spectra for graphene and Si-graphene: (a) survey, (b) C_1s_, (c) O_1s_, and (d) Si_2p_ spectra.

The surface characteristics of pristine graphene and Si-graphene were investigated by using XPS (Figure 3). The characteristic peak for C_1s_ was observed at 285.1 eV, and its intensity significantly decreased after surface modification (Figure 3b), while the characteristic peak of O_1s_ appeared at 532.6 eV, with its intensity increasing dramatically after surface modification (Figure 3c). A new peak was also observed at 102.5 eV after surface modification, which was ascribed to Si_2p_ (Figure 3d). The atomic C/O ratio calculated from the C_1s_ and the O_1s_ peaks in the XPS spectra decreased from 12.61 for pristine graphene to 2.87 for Si-graphene. These results can be explained by the fact that, after surface modification with the silane coupling agent, oxygen-containing functional groups, such as C=O, C–O, and Si–O, were introduced to the graphene surface,^28,44,45^ reducing the intensity of the C_1s_ peak and increasing the intensity of the O_1s_ and Si_2p_ peaks.

**Figure 4:**
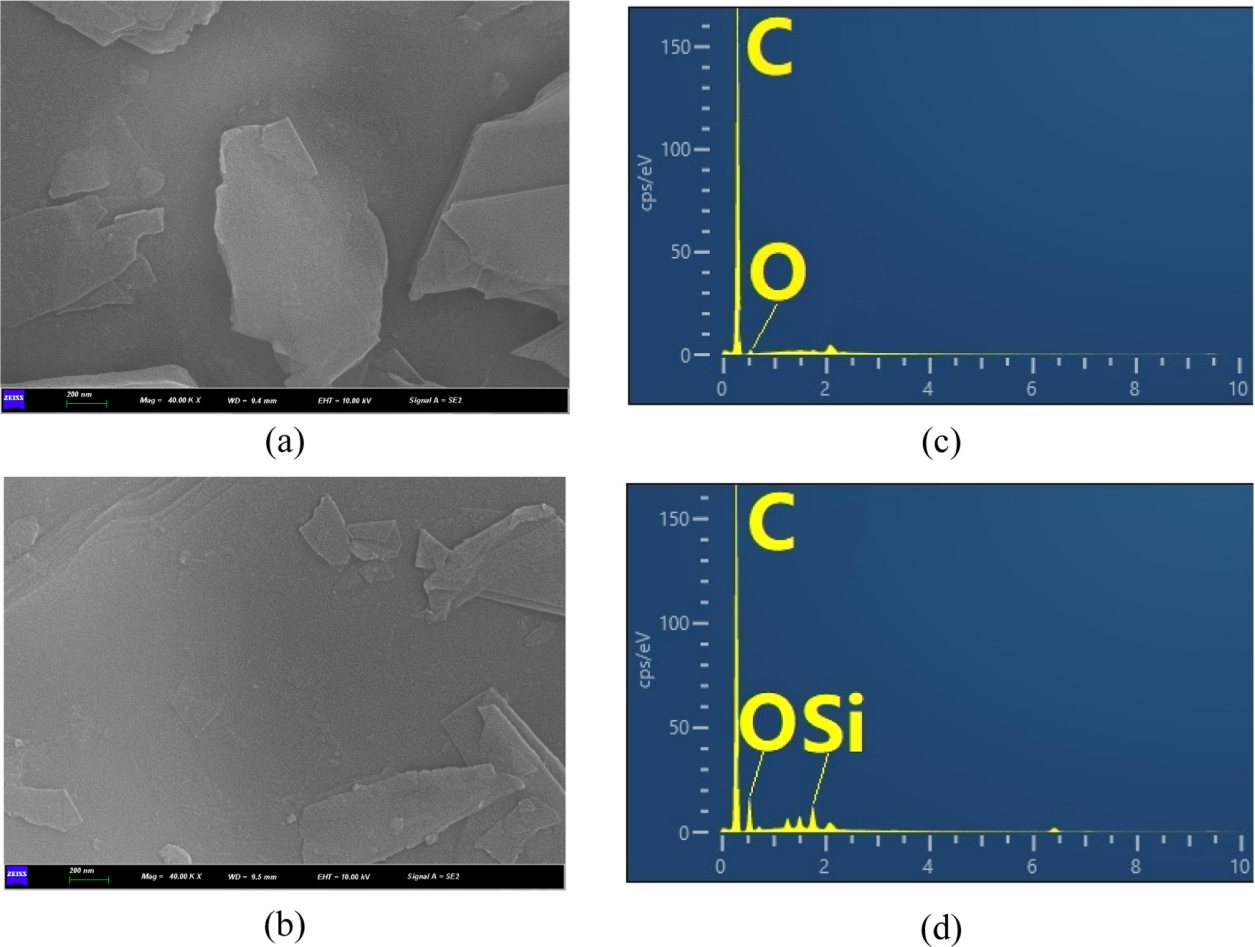
(a, b) SEM images of (a) graphene and (b) Si-graphene (magnification of 40,000; scale bar = 200 nm). (c, d) EDX maps of (c) graphene and (d) Si-graphene.

SEM–EDX analysis was also conducted to investigate the morphology of graphene before and after surface modification and to verify the presence of silicon on the graphene surface. Figure 4a,b presents the surface morphology of pristine graphene and Si-graphene, respectively. The pristine graphene exhibited a smooth surface, while small white particles appeared on the surface of Si-graphene, indicating the presence of organic functional groups after surface modification.

Figure 4c,d displays the EDX maps for pristine graphene and Si-graphene, respectively. The peaks for the pristine graphene at approximately 0.30 and 0.55 keV were attributed to carbon and oxygen, respectively, while a new silicon peak at approximately 1.68 keV appeared after surface modification. In particular, after surface modification, the carbon content decreased from 96.4 to 83.25%, and the oxygen and silicon content increased from 3.4 and 0% to 14.82 and 1.93%, respectively. Collectively, these results verify the successful surface modification of graphene using the silane coupling agent.

### 3.2 Electrical Properties

**Figure 5:**
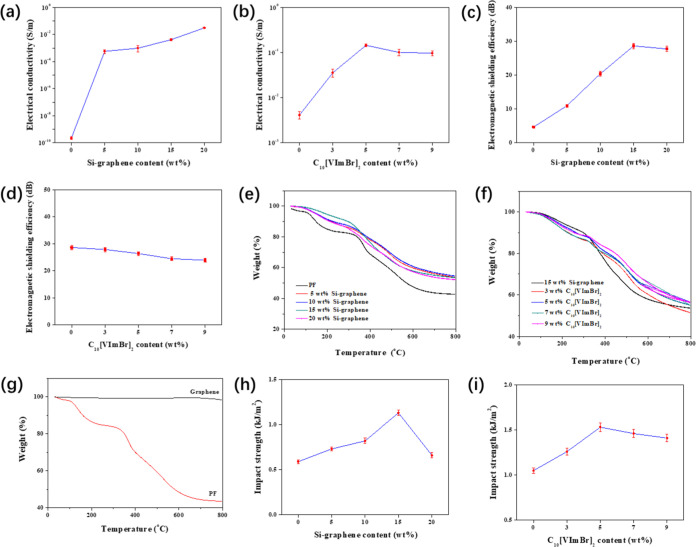
(a, b) Electrical conductivity of the (a) PF/Si-graphene and (b) PF/Si-graphene/C_10_[VImBr]_2_ composites. (c, d) Electromagnetic shielding efficiency of the (c) PF/Si-graphene and (d) PF/Si-graphene/C_10_[VImBr]_2_ composites. (e–g) TGA thermograms for the (e) PF/Si-graphene and (f) PF/Si-graphene/C_10_[VImBr]_2_ composites, and (g) graphene and PF. (h, i) Impact strength of the (h) PF/Si-graphene and (i) PF/Si-graphene/C_10_[VImBr]_2_ composites.

The electrical properties of the PF/Si-graphene and PF/Si-graphene/C_10_[VImBr]_2_ composites were investigated by using electrical conductivity measurements. Figure 5a presents the electrical conductivity of the PF/Si-graphene composites, which significantly increased with the addition of Si-graphene. Pristine PF had a low electrical conductivity of 2.3 × 10^–10^ S/m, classifying it as an insulating material. In contrast, the electrical conductivity of the PF/Si-graphene composites containing 15 and 20 wt % Si-graphene was 4.14 × 10^–3^ and 3.1 × 10^–2^ S/m, respectively, which was 1.8 × 10^7^ and 1.3 × 10^8^ times higher than that of pristine PF. This can be attributed to the high electrical conductivity and large surface area of graphene, which created electrically conductive pathways within the PF matrix, thus increasing the electrical conductivity of the PF/Si-graphene composites.^43,46,47^

The electrical conductivity of the PF/Si-graphene/C_10_[VImBr]_2_ composites as a function of the C_10_[VImBr]_2_ content is presented in Figure 5b. The electrical conductivity of the composites increased significantly with the addition of C_10_[VImBr]_2_. In particular, the electrical conductivity with 5 wt % C_10_[VImBr]_2_ was 0.145 S/m, which was 34 times higher than 15 wt % Si-graphene and 6.3 × 10^8^ times higher than pristine PF. The addition of ionically conductive C_10_[VImBr]_2_ to the PF-based polymer network retains the ionized state of the anions and cations and provides a bridge for electron transfer between the graphene layers, which promotes the formation of electrically conductive pathways within the PF matrix.^41,48^ Furthermore, the hydrophilic nature of the imidazole ring in C_10_[VImBr]_2_ facilitates the dispersion of graphene in the PF matrix. In the present study, this led to the formation of more electrically conductive pathways, thus increasing the electrical conductivity of the PF/Si-graphene/C_10_[VImBr]_2_ composites.^49,50^

### 3.3 Electromagnetic Shielding Efficiency

Figure 5c presents the electromagnetic shielding efficiency of the PF/Si-graphene composites as a function of the Si-graphene content. The electromagnetic shielding efficiency of the composites significantly increased with the addition of Si-graphene from 4.7 dB for pristine graphene to 28.64 and 27.73 dB for the composites with 15 and 20 wt % Si-graphene, respectively, representing a 509 and 490% increase. This was because the dispersion of graphene within the PF matrix improved after surface modification, which led to the formation of electrically conductive networks. When electromagnetic waves entered the composite, they were repeatedly reflected and absorbed between the graphene layers, thus improving the electromagnetic shielding efficiency of the PF/Si-graphene composites.^51,52^

The electromagnetic shielding efficiency of the PF/Si-graphene/C_10_[VImBr]_2_ composites decreased with an increase in the C_10_[VImBr]_2_ content (Figure 5d). In particular, the electromagnetic shielding efficiency of the PF/Si-graphene/C_10_[VImBr]_2_ composite with 9 wt % C_10_[VImBr]_2_ was 23.93 dB, which was 16% lower than that of the PF/Si-graphene composites. Thus, while the addition of C_10_[VImBr]_2_ can improve the electrical conductivity of the composites, it reduces the electromagnetic shielding efficiency. This can be explained by the fact that an ideal electromagnetic shielding material requires not only conductive components but also other properties (such as magnetism) to improve impedance matching.^53,54^

### 3.4 Thermal Stability

The thermal stability of the PF/Si-graphene and PF/Si-graphene/C_10_[VImBr]_2_ composites was investigated using TGA (Figure 5e,f, respectively). Two indicators of thermal stability—the initial decomposition temperature (i.e., the temperature at which 5% weight loss occurs; *T*_5%_) and the amount of char at 800 °C—were calculated from the TGA thermograms,^55,56^ and the results are summarized in Table 1.

**Table 1:** Thermal Stability of PF/Si-graphene and PF/Si-graphene/C_10_[VImBr]_2_ Composites

Si-graphene content (wt %)	C_10_[VImBr]_2_ content (wt %)	*T*_5%_ (°C)^a^	amount of char formation at 800 °C (%)^a^
0	0	112.4	42.7
5	0	153.1	53.8
10	0	152.0	54.5
15	0	154.2	53.6
20	0	147.2	52.3
15	3	156.2	51.4
15	5	157.4	55.0
15	7	168.3	56.3
15	9	177.0	56.6

a Note: *T*_5%_ and the amount of char at 800 °C were determined from TGA thermograms.

The *T*_5%_ of the PF/Si-graphene composites significantly increased with the addition of Si-graphene from 112.4 °C for pristine PF to 147.2–154.2 °C for the composites, an increase of 34.8–41.8 °C. These results can be explained by the higher temperature required for the 1% loss of weight (749.9 °C) and the larger residual mass of graphene at 800 °C (98.3%) compared with pristine PF (Figure 5g). Moreover, graphene sheets dispersed within the PF matrix acted as a physical barrier that slowed down the diffusion of pyrolysis products, thus increasing the thermal stability of the PF/Si-graphene composites.^57−59^ In addition, the char formation of the composites at 800 °C significantly increased with greater Si-graphene content due to the high residual mass of graphene.

The *T*_5%_ of the PF/Si-graphene/C_10_[VImBr]_2_ composites also increased with the addition of C_10_[VImBr]_2_ (Table 1), reaching 156.2–177.0 °C, which was 2.0–22.8 °C higher than that of the PF/Si-graphene composites and 43.8–64.6 °C higher than that of pristine PF. This was because the vinyl groups in C_10_[VImBr]_2_ self-polymerized or participated in the curing reaction for PF, thus increasing the cross-linking density of the PF/Si-graphene/C_10_[VImBr]_2_ composites. The dispersion of graphene within the PF matrix was also facilitated by the hydrophilic nature of the imidazole ring in C_10_[VImBr]_2_, and the graphene sheets limited the movement of the polymer chains via physical interlocking and interfacial adhesion, slowing the diffusion of the pyrolysis products during thermal decomposition.^49,60^ The addition of C_10_[VImBr]_2_ had little effect on the char formation of the PF/KH-graphene/C_10_[VImBr]_2_ composites at 800 °C.

### 3.5 Impact Strength

The impact strengths of the PF/Si-graphene and PF/Si-graphene/C_10_[VImBr]_2_ composites were also investigated. Figure 5h shows that the impact strength of the PF/Si-graphene composites increased with the addition of a Si-graphene content. Pristine PF, which is classified as a brittle material, has an impact strength of 0.59 kJ/m^2^ at room temperature.^55^ In contrast, the impact strength of the composites with 15 wt % Si-graphene was 1.13 kJ/m^2^, which was 91% higher than that of pristine PF. This was attributed to the improved dispersion of graphene within the PF matrix after surface modification. The graphene induced the formation of numerous microcracks in the PF matrix, which absorbed the external energy from the impact force, thus improving the impact strength of the PF/Si-graphene composites.^61^

Figure 5i shows that the addition of C_10_[VImBr]_2_ improved the impact strength of the PF/Si-graphene/C_10_[VImBr]_2_ composites. At 5 wt % C_10_[VImBr]_2_, the impact strength was 1.53 kJ/m^2^, which was 45% higher than that of the PF/Si-graphene composites and 159% higher than that of pristine PF. This was attributed to the strong intermolecular interaction between C_10_[VImBr]_2_ and the PF matrix due to the formation of a stable polar conjugate structure via the self-polymerization of C_10_[VImBr]_2_ or the copolymerization of C_10_[VImBr]_2_ with PF.^62^

### 3.6 Morphology

**Figure 6:**
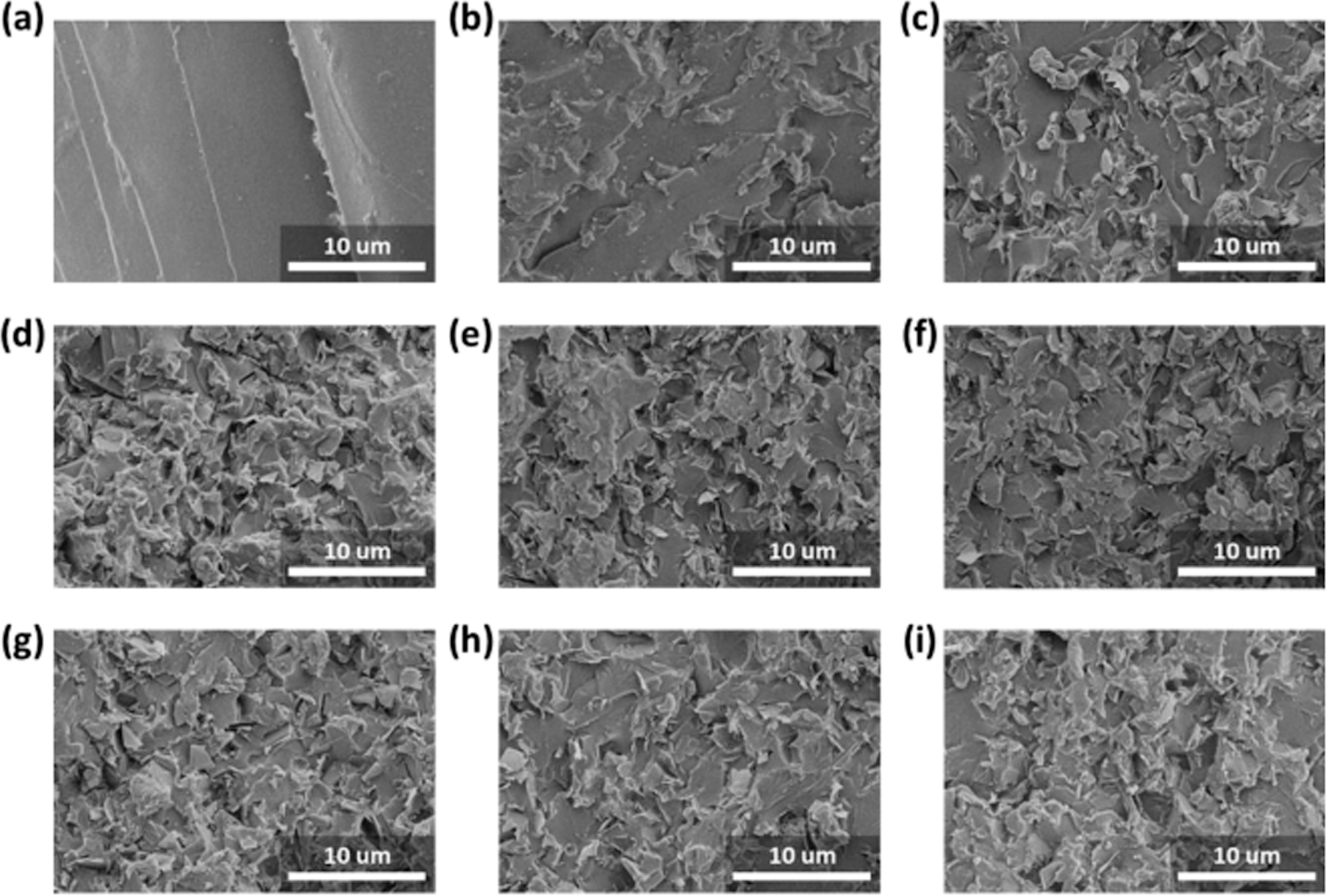
SEM micrographs of the PF/Si-graphene composites and PF/Si-graphene/C_10_[VImBr]_2_ composites: (a) pristine PF, (b) 5 wt % Si-graphene, (c) 10 wt % Si-graphene, (d) 15 wt % Si-graphene, (e) 20 wt % Si-graphene, (f) 15 wt % Si-graphene + 3 wt % C_10_[VImBr]_2_, (g) 15 wt % Si-graphene + 5 wt % C_10_[VImBr]_2_, (h) 15 wt % Si-graphene + 7 wt % C_10_[VImBr]_2_, and (i) 15 wt % Si-graphene +9 wt % C_10_[VImBr]_2_ (magnification: × 2000).

The morphology of the PF/Si-graphene and PF/Si-graphene/C_10_[VImBr]_2_ composites after the impact strength tests was investigated by using SEM. Figure 6a–e presents SEM images of the fracture surface of the PF/Si-graphene composites. As shown in Figure 6a, pristine PF had a mirror-like morphology and ordered cracking behavior, indicating brittle deformation prior to fracture.^63^ In contrast, the PF/Si-graphene composites exhibit a relatively rough morphology with numerous microcracks, indicating that they absorbed more external energy^64,65^ (Figure 6b–e).

Figure 6f–i presents the morphology of the PF/Si-graphene/C_10_[VImBr]_2_ composites according to the C_10_[VImBr]_2_ content. These composites exhibited a rough morphology with numerous tortuous microcracks, which explained their high impact strength,^66^ as revealed by the SEM images in Figure 6f–i.

## 4 Conclusions

In the present work, the synergistic effect of silanized graphene and C_10_[VImBr]_2_ on the electrical properties, electromagnetic shielding efficiency, thermal stability, impact strength, and morphology of PF/Si-graphene and PF/Si-graphene/C_10_[VImBr]_2_ composites was investigated. It was found that the electrical conductivity of the composites increased from 2.3 × 10^–10^ to 4.14 × 10^–3^ S/m with an increase in Si-graphene from 0 to 20 wt % due to the high electrical conductivity and large surface area of graphene. When 5 wt % C_10_[VImBr]_2_ was added, the electrical conductivity improved further to 0.145 S/m because C_10_[VImBr]_2_ acted as a bridge for electron transfer between the graphene layers and promoted the formation of electrically conductive pathways within the PF matrix. The electromagnetic shielding efficiency of the composites reached 28.64 dB with 15 wt % graphene, which was 509% higher than that of pristine PF due to the repeated reflection and absorption of electromagnetic waves between the graphene layers dispersed in the PF matrix. The *T*_5%_ of PF/Si-graphene and PF/Si-graphene/C_10_[VImBr]_2_ composites was 34.8–41.8 and 43.8–64.6 °C higher than that of pristine PF, respectively, due to graphene’s high thermal stability and its role as a physical barrier, combined with a high cross-linking density. The impact strength of the composites increased from 0.59 to 1.13 kJ/m^2^ (a 91% increase) with an increase in the Si-graphene content from 0 to 15 wt %, which was due to the absorption of external energy by a large number of microcracks caused by the uniformly dispersed graphene in the PF matrix. It further increased to 1.53 kJ/m^2^ (45% increase) with the addition of 5 wt % C_10_[VImBr]_2_, which was due to the high intermolecular interaction between C_10_[VImBr]_2_ and the PF matrix. The SEM images of the PF/Si-graphene/C_10_[VImBr]_2_ composites revealed a rough morphology with numerous microcracks. The outcomes of this study demonstrate that PF/Si-graphene/C_10_[VImBr]_2_ ternary composites with high electrical conductivity, electromagnetic shielding efficiency, thermal stability, and impact strength can be successfully employed for electromagnetic shielding applications.
